# Supplementary figures and images for: Evaluation of a toxoid fusion protein vaccine produced in plants to protect poultry against necrotic enteritis
Source: PeerJ. 2019 Mar 28;7:e6600. doi: 10.7717/peerj.6600 (PMC6441560; doi:10.7717/peerj.6600)

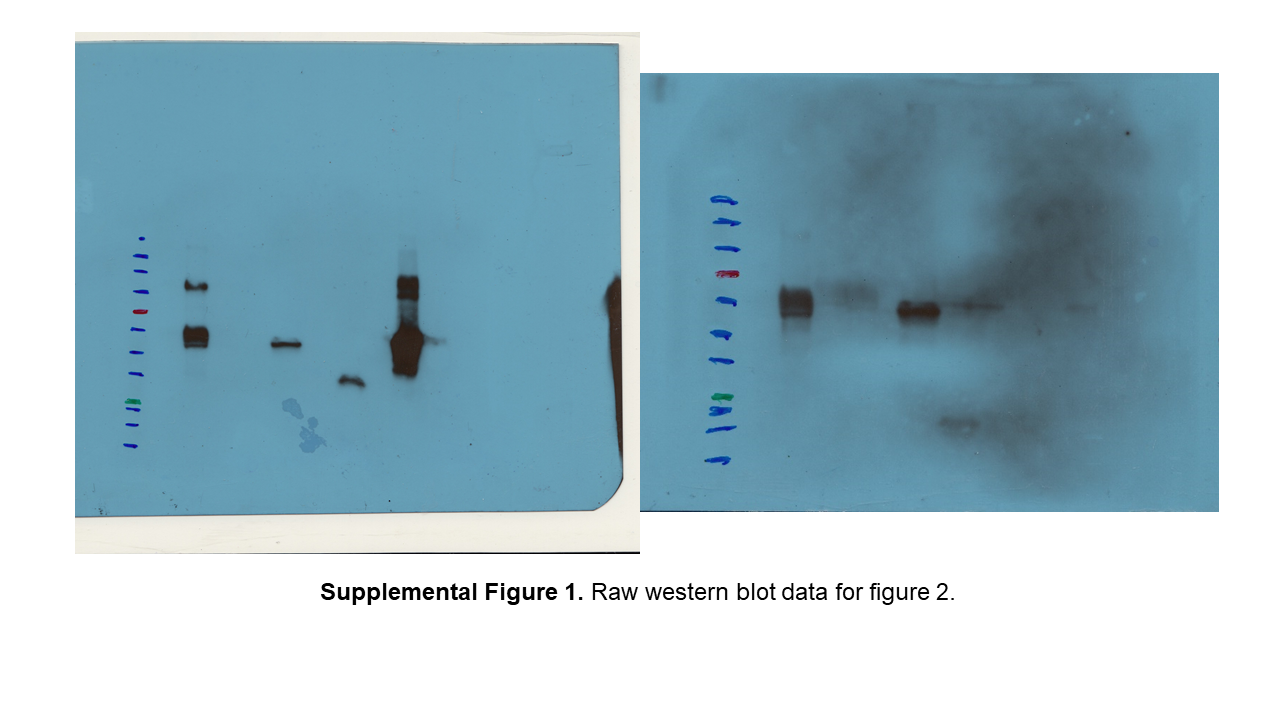

Supplement: Supplemental Information 1 [file peerj-07-6600-s001.png]

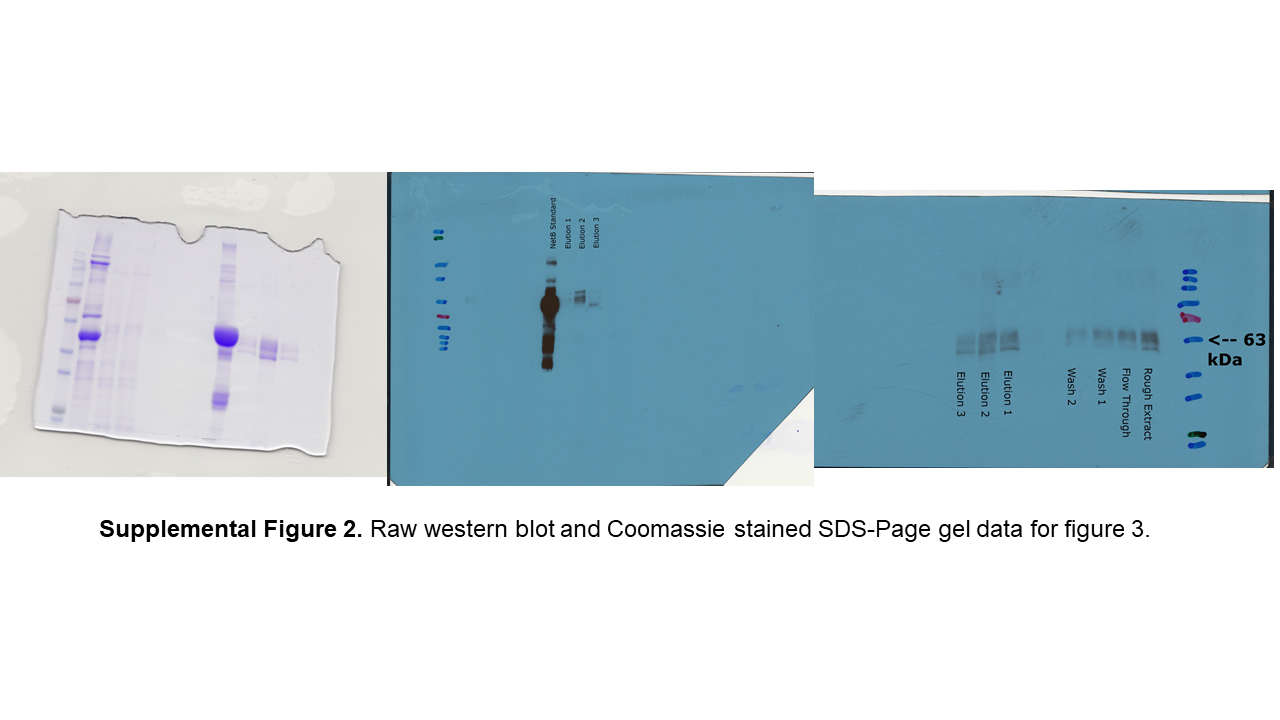

Supplement: Supplemental Information 2 [file peerj-07-6600-s002.png]
