# Supplementary material for: Evaluation of a toxoid fusion protein vaccine produced in plants to protect poultry against necrotic enteritis
Source: PeerJ. 2019 Mar 28;7:e6600. doi: 10.7717/peerj.6600 (PMC6441560; doi:10.7717/peerj.6600)
Supplement: Supplemental Information 3 — DNA sequences and encoded amino acid sequences for the 6His-PlcC-NetB fusion protein either with or without ER-targeting signal peptide. [file peerj-07-6600-s003.docx]

**6His-PlcC-NetB fusion protein,ER targeted (with signal peptide)**

ATGGCTAACAAgCAcCTcTCaTTGTCTCTcTTCCTtGTGCTcCTTGGTCTTTCTGCTTCTCTTGCTTCTGGTCACCATCACCATCATCACggatccGACCCaTCCGTGGGaAACAACGTtAAgGAgCTtGTGGCTTACATCTCCACTtctGGaGAgAAgGACGCTGGaACCGACGAtTACATGTAcTTCGGtATCAAgACCAAGGAtGGaAAgACTCAAGAATGGGAgATGGACAAtCCaGGtAACGACTTCATGGCTGGtAGCAAgGAtACTTAcACTTTCAAgTTgAAAGACGAgAACCTtAAgATcGACGACATCCAgAACATGTGGATTaggAAACGTAAgTAcACCGCcTTCCCaGACGCTTAcAAGCCtGAgAACATCAAGGTtATCGCtAACGGaAAgGTGGTtGTtGACAAGGAtATCAACGAGTGGATTTCtGGaAACTCCACTTAcAACATCAAAggaggttctggtggatcaggaggtccatctggaggttctggaggatccGAgCTtAACGACATCAACAAgATTGAGCTtAAgAACCTctcCGGaGAgATCATCAAgGAgAACGGtAAGGAgGCTATcAAgTAcACTTCttccGACACCGCTTCCCAcAAgGGaTGGAAGGCcACTCTttctGGaACCTTCATcGAAGACCCtCATTCtGACAAGAAgACTGCTttgCTtAACCTtGAAGGaTTcATCCCaTCtGACAAACAGATcTTCGGaTCTAAgTAcTACGGaAAgATGAAgTGGCCtGAgACTTAcaggATcAAcGTGAAgAGCGCTGACGTtAACAAcAACATCAAgATCGCcAACTCtATTCCGAAgAAcACTATCGACAAgAAgGACGTGTCCAATTCtATcGGtTAcTCCATCGGaGGTAACATCTCtGTtGAgGGtAAgACTGCTGGtGCTGGaATCAACGCTTCtTAcAACGTtCAgAACACTATCtccTATGAgCAACCtGACTTCagaACCATTCAgaggAAgGACGATGCtAACCTtGCATCCTGGGACATCAAATTCGTTGAGACTAAGGACGGaTAcAACATCGACTCCTAcCATGCTATcTATGGCAACCAgCTcTTCATGAAgagcagattgTAcAACAATGGtGACAAgAACTTCACCGACGATaggGACCTcTCCACCttgATcTCtGGtGGaTTCTCtCCaAACATGGCTCTtGCcttgACCGCtCCTAAgAAcGCTAAgGAgTCaGTGATCATCGTtGAATAcCAgaggTTCGACAACGACTATATcCTtAAcTGGGAgACTACTCAAGCTagaGGaACtAACAAgCTTTCtTCAACCtccGAgTAcAACGAgTTTATGTTCAAgATCAACTGGCAgGACCAcAAgATCGAATAcTATCTtTAA

MANKHLSLSLFLVLLGLSASLASGHHHHHHGSDPSVGNNVKELVAYISTSGEKDAGTDDYMYFGIKTKDGKTQEWEMDNPGNDFMAGSKDTYTFKLKDENLKIDDIQNMWIRKRKYTAFPDAYKPENIKVIANGKVVVDKDINEWISGNSTYNIKGGSGGSGGPSGGSGGSELNDINKIELKNLSGEIIKENGKEAIKYTSSDTASHKGWKATLSGTFIEDPHSDKKTALLNLEGFIPSDKQIFGSKYYGKMKWPETYRINVKSADVNNNIKIANSIPKNTIDKKDVSNSIGYSIGGNISVEGKTAGAGINASYNVQNTISYEQPDFRTIQRKDDANLASWDIKFVETKDGYNIDSYHAIYGNQLFMKSRLYNNGDKNFTDDRDLSTLISGGFSPNMALALTAPKNAKESVIIVEYQRFDNDYILNWETTQARGTNKLSSTSEYNEFMFKINWQDHKIEYYL

**6His-PlcC-NetB fusion protein, cytoplasm targeting(no signal peptide)**

ATGGCTCACCATCACCATCATCACggatccGACCCaTCCGTGGGaAACAACGTtAAgGAgCTtGTGGCTTACATCTCCACTtctGGaGAgAAgGACGCTGGaACCGACGAtTACATGTAcTTCGGtATCAAgACCAAGGAtGGaAAgACTCAAGAATGGGAgATGGACAAtCCaGGtAACGACTTCATGGCTGGtAGCAAgGAtACTTAcACTTTCAAgTTgAAAGACGAgAACCTtAAgATcGACGACATCCAgAACATGTGGATTaggAAACGTAAgTAcACCGCcTTCCCaGACGCTTAcAAGCCtGAgAACATCAAGGTtATCGCtAACGGaAAgGTGGTtGTtGACAAGGAtATCAACGAGTGGATTTCtGGaAACTCCACTTAcAACATCAAAggaggttctggtggatcaggaggtccatctggaggttctggaggatccGAgCTtAACGACATCAACAAgATTGAGCTtAAgAACCTctcCGGaGAgATCATCAAgGAgAACGGtAAGGAgGCTATcAAgTAcACTTCttccGACACCGCTTCCCAcAAgGGaTGGAAGGCcACTCTttctGGaACCTTCATcGAAGACCCtCATTCtGACAAGAAgACTGCTttgCTtAACCTtGAAGGaTTcATCCCaTCtGACAAACAGATcTTCGGaTCTAAgTAcTACGGaAAgATGAAgTGGCCtGAgACTTAcaggATcAAcGTGAAgAGCGCTGACGTtAACAAcAACATCAAgATCGCcAACTCtATTCCGAAgAAcACTATCGACAAgAAgGACGTGTCCAATTCtATcGGtTAcTCCATCGGaGGTAACATCTCtGTtGAgGGtAAgACTGCTGGtGCTGGaATCAACGCTTCtTAcAACGTtCAgAACACTATCtccTATGAgCAACCtGACTTCagaACCATTCAgaggAAgGACGATGCtAACCTtGCATCCTGGGACATCAAATTCGTTGAGACTAAGGACGGaTAcAACATCGACTCCTAcCATGCTATcTATGGCAACCAgCTcTTCATGAAgagcagattgTAcAACAATGGtGACAAgAACTTCACCGACGATaggGACCTcTCCACCttgATcTCtGGtGGaTTCTCtCCaAACATGGCTCTtGCcttgACCGCtCCTAAgAAcGCTAAgGAgTCaGTGATCATCGTtGAATAcCAgaggTTCGACAACGACTATATcCTtAAcTGGGAgACTACTCAAGCTagaGGaACtAACAAgCTTTCtTCAACCtccGAgTAcAACGAgTTTATGTTCAAgATCAACTGGCAgGACCAcAAgATCGAATAcTATCTtTAA

MAHHHHHHGSDPSVGNNVKELVAYISTSGEKDAGTDDYMYFGIKTKDGKTQEWEMDNPGNDFMAGSKDTYTFKLKDENLKIDDIQNMWIRKRKYTAFPDAYKPENIKVIANGKVVVDKDINEWISGNSTYNIKGGSGGSGGPSGGSGGSELNDINKIELKNLSGEIIKENGKEAIKYTSSDTASHKGWKATLSGTFIEDPHSDKKTALLNLEGFIPSDKQIFGSKYYGKMKWPETYRINVKSADVNNNIKIANSIPKNTIDKKDVSNSIGYSIGGNISVEGKTAGAGINASYNVQNTISYEQPDFRTIQRKDDANLASWDIKFVETKDGYNIDSYHAIYGNQLFMKSRLYNNGDKNFTDDRDLSTLISGGFSPNMALALTAPKNAKESVIIVEYQRFDNDYILNWETTQARGTNKLSSTSEYNEFMFKINWQDHKIEYYL
