# Supplementary material for: Evaluation of a toxoid fusion protein vaccine produced in plants to protect poultry against necrotic enteritis
Source: PeerJ. 2019 Mar 28;7:e6600. doi: 10.7717/peerj.6600 (PMC6441560; doi:10.7717/peerj.6600)
Supplement: Supplemental Information 4 — Underlined sequences indicate engineered restriction sites used for cloning. [file peerj-07-6600-s004.docx]

**Table S1**. Primers used in this study*

| Primer | DNA sequence |
| --- | --- |
| PlcC-Sac-R | cccgagctcctaTTTGATGTTgTAAGTGGAGTTtCC |
| 6H-Xba-F | gacTCTAGAacaATGGCTCACCATCACCATCATCAC |
| Plc_pqe_f1 | ACATGGATCCGACCCGTCCGTGGGCAACAAC |
| Plc_pqe_r2 | ACATGTCGACGTTTCCTAAGCTTCTATTTG |
| netB_pqe_f3 | GGGATCCAGCGAACTGAACGAC |
| netB_Pqe_r4 | GTCGACGCTTTTACAGATAATATTC |

*Underlined sequences indicate engineered restriction sites used for cloning.
